# Culture-Negative *Streptococcus suis* Infection Diagnosed by Metagenomic Next-Generation Sequencing

**DOI:** 10.3389/fpubh.2019.00379

**Published:** 2019-12-17

**Authors:** Yuanyuan Dai, Li Chen, Wenjiao Chang, Huaiwei Lu, Peng Cui, Xiaoling Ma

**Affiliations:** ^1^Department of Clinical Laboratory, First Affiliated Hospital of University of Science and Technology of China, Hefei, China; ^2^Infectious Disease Research and Development, Beijing Genomics Institute-Shanghai, Shanghai, China

**Keywords:** *Streptococcus suis*, bacteria, streptococcal toxic shock syndrome, metagenomic next-generation sequencing, blood culture

## Abstract

**Background:** *Streptococcus suis* is a zoonotic pathogen that can cause severe infections such as meningitis and septicemia in both swine and humans. Rapid and accurate identification of the causative agent is very important for guiding clinical choices in administering countermeasures.

**Case Report:** Here, we report a case of fatal *S. suis* infection in a patient who worked as a butcher in China. The 59-year-old man, who had previously undergone splenectomy, injured his finger while processing pork and developed severe sepsis. While blood cultures were negative following antibiotic treatment, *S. suis* was determined to be the causative agent by metagenomic next-generation sequencing (mNGS) and Sanger sequencing.

**Conclusion:** Identification of etiological agents using techniques such as blood culture prior to antibiotic treatment is very important. mNGS may represent a useful method for diagnosis of infectious diseases, especially post-antibiotic treatment.

## Background

*Streptococcus suis* is a major swine pathogen, and typically colonizes the nasal cavities, tonsils, and upper respiratory, genital, and gastrointestinal tracts (1, 2). *Streptococcus suis* has recently been recognized as a zoonotic pathogen that may cause infections in humans in occupational contact with pigs or pork (3, 4). Meningitis is the most frequent clinical presentation, followed by septicemia, and arthritis (4). *Streptococcus suis* infection has nonspecific clinical symptoms in the early stages and progresses rapidly. Mortality rates (0–33.3%) are low compared with meningitis caused by other pathogens (5–12). However, toxic shock-like syndrome caused by this pathogen has a high mortality rate in China (62 and 50%) and Thailand (80%) (6–8). Considering the severity of *S. suis* infection in humans, developing methods for rapid, and accurate identification of the causative agent is critically important for timely and efficient treatment.

In recent years, metagenomic next-generation sequencing (mNGS) has been applied in medical microbiology for the unbiased detection of viruses, bacteria, fungi, and eukaryotic parasites in clinical samples (13). mNGS only requires a small amount of genetic material obtained directly from patient samples for whole genome sequencing without bacterial culture. Pathogens are identified by linking sequencing reads to a catalog of clinical pathogens, thereby listing the potential causative agents (14). mNGS hold enormous promise to improve diagnosis of infectious diseases, especially in immunocompromised and critically ill patients (15). In this report, we applied mNGS to rapidly identify that *S. suis* was the causative agent of severe sepsis in a 59-year-old man who had previously undergone splenectomy.

## Case Presentation

In September 2018, a 59-year-old butcher injured his finger while working with pork. He was admitted to the local hospital in Tongling, Anhui Province, Eastern China. His symptoms included a high fever for 2 days, vomiting, myasthenia of the limbs and somnolence. In the local hospital, he was treated by debridement and anti-infection therapy with intravenous linezolid and imipenem, but the disease progressed quickly. The patient was transferred to our hospital for further treatment. On arrival, the patient showed subcutaneous ecchymosis and was diagnosed with septic shock. His vital signs were as follows: blood pressure 98/61 mmHg (dobutamine and norepinephrine were given to maintain blood pressure), heart rate 128 beats/min, respiratory rate 20 breaths/min, and body temperature 38.6°C. Lung sounds were clear, but bowel sounds were reduced. His laboratory data were as follows: leukocytosis (white blood cell count, 17.40 × 10^9^/L; neutrophils, 88.7%; lymphocytes, 7.3%; monocytes, 3.8%), thrombocytopenia (platelet count, 14 × 10^9^/L), coagulation function (PT, 61.7 s; PT-INR, 7.23; APTT, 66.9 s; FIB, 0.32 g/L; TT, 159.5 s), inflammatory markers (CRP, 107.98 mg/L; PCT, 100.0 ng/mL), cardiac serum markers (CK, 652 IU/L; CK-MB, 82.05 IU/L; LDH, 10,750 IU/L; MYO, 1298.0 μg/L; TnI, 51 μg/L; BNP, >35,000 pg/mL), high urea nitrogen, and creatinine (urea nitrogen, 30.9 mg/dL; creatinine, 234.4 μmol/L), hyperbilirubinemia (total bilirubin; 183.8 μmol/L), and metabolic acidosis (pH, 7.03; pO_2_, 79 mmHg; pCO_2_, 39.5 mmHg; Lac >15 mmol/L). The patient was diagnosed with severe septicemia and multi-organ dysfunction with acute respiratory, renal and hepatic failure, as well as heart malfunction with disseminated intravascular coagulation (DIC). Meningitis were not observed. Vancomycin and ceftriaxone were administered as empirical antibiotics after taking a blood sample for bacterial culture and mNGS. The patient was then admitted to the intensive care unit for close monitoring. Mechanical ventilation was started because of severe respiratory failure.

The mNGS-based detection of pathogens in the patient's blood sample was approved by his son and the patient's physician. His blood sample was immediately sent to BGI-Shanghai. Briefly, the blood sample was centrifuged at 1,600 × g for 10 min at 4°C. DNA was extracted from 300 μL of plasma using the QIAamp DNA Mini Kit (Qiagen). The extracted DNA was fragmented by sonication to yield 200–500 bp fragments. DNA libraries were then constructed by end-repair, dA-tailing, adapter ligation and PCR amplification. An Agilent 2100 Bioanalyzer was used for quality control of the DNA libraries. The final libraries were sequenced using the BGISEQ-100 platform (16). Raw data were preprocessed by removing low quality reads, residual adapters and short reads. Reads that mapped to a human reference genome (hg19) using Burrows-Wheeler Alignment (17) were removed. Subsequently, the remaining sequences were aligned to bacterial, viral, and fungal databases(NCBI; ftp://ftp.ncbi.nlm.nih.gov/genomes). The depth and coverage of each species were calculated using the SOAP website (http://soap.genomics.org.cn/).

**Figure 1:**
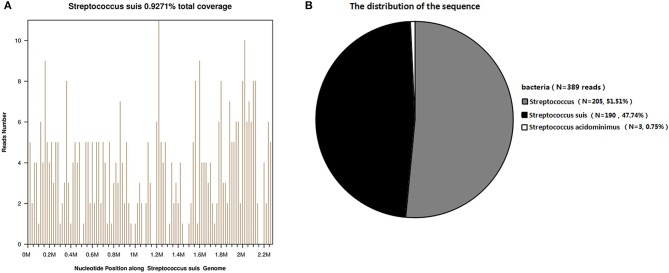
Diagnosis of *S. suis* infection using mNGS. **(A)** The majority of reads mapped to the *S. suis* genome, with coverage of 0.9271%. **(B)** A total of 190 sequence reads corresponded to *S. suis*, accounting for 47.74% of microbial reads.

After 2 days, the blood mNGS report detected a total of 190 (out of 398) unique *S. suis* sequence reads, representing 0.9276% of the nucleotide sequence coverage (Figure 1). Blood bacterial cultures were negative after 5 days of incubation. Considering that antibiotics covered Gram-positive cocci, the patient's antibiotic treatment was not changed. His condition progressively deteriorated and he died 23 days after his admission to hospital.

## Discussion

*Streptococcus suis* is a Gram-positive, capsulated, hemolytic, facultative anaerobic coccus. We report a fatal case of *S. suis* infection with rapidly progressing severe septic shock. Although we obtained two-site blood cultures when the patient was first admitted to hospital, both cultures were negative. This may be related to the patient's previous experience of antibiotics in the local hospital.

Clinical signs and symptoms, related epidemiological data, and laboratory examinations play important roles in the diagnosis of human *S. suis* infection. mNGS, as a new technology, is an important additional to the diagnostic toolbox for infectious diseases of unknown etiology (13). Compared with traditional pathogen detection methods, mNGS can detect multiple pathogen gene sequences simultaneously to achieve unbiased and comprehensive screening. In recent years, there has been strong interest in using mNGS for identification of the pathogens responsible for bacterial meningitis (18, 19), septicemia (20), and infective endocarditis (21). In addition, mNGS only requires fragments of pathogen DNA obtained directly from patient samples rather than living pathogens. The results in this case showed that *S. suis* could be detected by mNGS even following antibiotic therapy. Thus, mNGS is generally more sensitive for identifying organisms than culture-based techniques (22–24). To our knowledge, this is the first report of direct detection of *S. suis* from clinical samples using mNGS.

Major risk factors associated with *S. suis* infection include pig-related occupations, exposure to pig or pork products, skin injury especially during work with pork products, and male gender (25–27). Our patient was a 59-year-old man who injured his finger while processing of pork, and thus multiple common risk factors for *S. suis* infection were present. Other key characteristics that make patients vulnerable to *S. suis* infection include previous splenectomy, alcoholic liver disease, diabetes, heart disease, or corticosteroid use and resulting immunosuppression. Our patient had previously undergone splenectomy. Splenectomized patients are at significant risk of infection because the spleen is the largest accumulation of lymphoid tissue in the human body. In the absence of these tissues, the ability to fight off the pathogens is severely diminished. It is well-known that splenectomized patients are more likely to develop severe infection, a condition known as overwhelming post splenectomy infection (OPSI) syndrome (28). The incubation period of our case was short and disease progression was rapid, with multisystem dysfunction and DIC, thus meeting the clinical definition of OPSI. OPSI is a serious complication of asplenia and is associated with encapsulated organisms (29). After the occurrence of *S. suis* infection in splenectomized patients, it is difficult to remove the invasive pathogens quickly and effectively. *Streptococcus suis* can cause extensive tissue cell damage, systemic capillary leakage, and multiple organ failure in a very short period of time (30–32).

## Conclusions

Here, we reported the application of mNGS to identify *S. suis* in a patient whose blood bacterial cultures were negative. While empirical use of antibiotics might inhibit bacterial proliferation, *S. suis* genetic material could still be detected. In China, empirical use of antibiotics is very common, making it difficult to diagnose pathogens. Thus, mNGS may represent an alternative method for diagnosis of pathogens, especially post-antibiotic treatment.

## Data Availability Statement

The datasets for this study can be found in the EMBL-EBI https://www.ebi.ac.uk/ena/data/view/PRJEB35295 under accession number PRJEB35295.

## Ethics Statement

We obtained written informed consent from the patient's family members in accordance with the principles laid out in the Declaration of Helsinki. Family members gave written informed consent to the study and for publication of clinical information, images and sequencing data. The study was exempt from ethical approval procedures as a case report of a single patient. As per the Institutional Ethical Committees of the First Affiliated Hospital of the University of Science and Technology of China, no pre-approval was required for the publication of a case report of a single patient.

## Author Contributions

XM, YD, and HL contributed conception and design of the study. LC organized the database. PC performed the statistical analysis. YD wrote the first draft of the manuscript. YD, LC, and WC wrote sections of the manuscript. All authors contributed to manuscript revision, read, and approved the submitted version.

### Conflict of Interest

PC was employed by Beijing Genomics Institute-Shanghai. The remaining authors declare that the research was conducted in the absence of any commercial or financial relationships that could be construed as a potential conflict of interest.
